# Correlation Analysis Between Ovarian Reserve and Thyroid Hormone Levels in Infertile Women of Reproductive Age

**DOI:** 10.3389/fendo.2021.745199

**Published:** 2021-09-27

**Authors:** Jie Wu, Ying-jie Zhao, Min Wang, Ming-qiang Tang, Yao-fang Liu

**Affiliations:** ^1^ Department of Clinical Laboratory, Fushun Maternal and Child Health Hospital, Fushun, China; ^2^ Department of Reproductive Technology, The Affiliated Hospital of Southwest Medical University, Luzhou, China

**Keywords:** infertility, hypothyroidism, Anti-Müllerian hormone, ovarian reserve, retrospective study 3

## Abstract

**Objective:**

To analyze the correlation between ovarian reserve and thyroid function in women with infertility.

**Methods:**

Retrospective analysis of the data of 496 infertility patients who visited the clinic between January 2019 and December 2020. According to the TSH level, it is grouped into <2.5 mIU/L, 2.5~4.0mIU/L and ≥4.0 mIU/L or according to the positive/negative thyroid autoimmune antibody. The relationship was assessed through the ovarian reserve, thyroid function, and anti-Müllerian hormone (AMH) levels in infertile patients. On the other hand, the patients are divided into groups according to age (≤29 years old, 30-34 years old and ≥35 years old), basic FSH (<10 IU/L and ≥10 IU/L), and AMH levels. The ovarian reserve was evaluated through the AMH and the antral follicle count (AFC).

**Results:**

The average age of the patients was 30.31 ± 4.50 years old, and the average AMH level was 5.13 ± 4.30 ng/mL. 3.63% (18/496) of patients had abnormal TSH levels (normal: 0.35-5.5 mIU/L), the positive rate of thyroid peroxidase antibody (TPOAb) was 14.52% (72/496), the positive rate of anti-thyroglobulin antibody (TgAb) was 16.94% (84/496), and the positive rate of TPOAb and TgAb was 10.48% (52/496). After grouping according to TSH level or thyroid autoimmune antibody positive/negative grouping, the analysis found that there was no statistical significance in age, AMH level and basic FSH level among the groups (P>0.05). There were no significant differences in the levels of TSH, FT3, and FT4 among different ages, AMH, and FSH levels (P>0.05).

**Conclusion:**

There is no significant correlation between ovarian reserve and thyroid function in infertile women.

## Introduction

The symptoms of thyroid dysfunction are often associated with the change of thyroid hormones involved in all phases of reproduction and fertility (1). According to the current guidelines, the laboratory test of pituitary hormone thyrotropin (TSH) is the most sensitive and specific marker for the diagnosis and management of thyroid dysfunction (2, 3). The hypothalamus-pituitary-thyroid axis is most relevant to thyroid function. The thyrotropin-releasing hormone (TRH) is secreted from the hypothalamus and reaches the anterior pituitary within the central nervous system. Activation of TRH receptors stimulates the release of thyroid-stimulating hormone (TSH), which activates its receptors on the thyroid gland’s follicular cells. It leads to increased cellular uptake of iodine from the blood and synthesis of thyroglobulin and secretion into the blood stream of triiodothyronine (T3) and thyroxine (T4) *via* activation of the enzyme thyroid peroxidase (TPO) (4).

Ovarian reserve function can reflect women’s endocrine function and fertility (5, 6) and is often related to age, and this function will gradually decrease (7). The diminished ovarian reserve (DOR) is defined by a reduced reproductive potential with a poor response to ovarian stimulation. Some young women still have DOR but the cause and mechanism of which are unknown. Previous studies have shown that TSH levels in infertile women were higher than those in normal fertile women (8, 9). Moreover, elevated serum TSH levels were associated with DOR in infertile patients (10). The hypothalamic-pituitary-ovarian and the hypothalamic-pituitary-thyroid axis have mutual regulation effects, such as the gonads of patients with polycystic ovary syndrome. The abnormal thyroid function may cause menstrual disorders and infertility (11, 12). It has been reported that hypoovarian reserve is related to increased TSH and thyroid autoimmune antibodies (13). On the contrary, no significant differences were observed in the prevalence of hypothyroidism between thyroid autoimmunity (TAI) and DOR (14). However, researchers have not yet determined whether the levels of TSH are associated with DOR.

Anti-Müllerian hormone (AMH) is a hormone produced by granulosa cells of early developing follicles. The serum AMH levels were closely correlated with the number of primordial follicles; therefore, AMH is a suitable biomarker for predicting ovarian function in premenopausal female patients. Therefore, it is equally important to determine whether ovarian function may be affected by impaired thyroid function in infertile patients. This study evaluated the relationship between the ovarian reserve, thyroid function, and AMH levels in infertile patients and may provide new ideas for evaluating DOR-related factors.

## Materials and Methods

### Patient Enrollment

Between January 2019 to December 2020, 496 consecutive Chinese women who enrolled the Affiliated Hospital of Southwest Medical University (Luzhou, China) and Fushun Maternal and Child Health Hospital (Fushun, China), respectively, and were diagnosed as infertile according to the diagnostic criteria shown below were recruited for participation in this study. Inclusion criterianormal sex life without contraception and have not been pregnant for more than 12 months. Exclusion criteria: (a) patients with polycystic ovary syndrome; (b) the patients who had a previous history of thyroid disorders, or presence of goiter and/or nodules, or thyroid surgery; (c) a history of hypothalamic and pituitary diseases; (d) with autoimmune diseases, diabetes, and adrenal gland dysfunction; (e) patients with history of disease and chromosomal abnormalities; (f) factors that adversely impact thyroid hormone and ovarian function. We measured thyroid-related hormone and serum AMH levels.

Serum levels of luteinizing hormone, follicle stimulating hormone, estradiol, progesterone, prolactin and testosterone were analyzed at 2–5 days of the menstrual cycle to screen for infertile patients.

Using all available data on DOR, we selected all patients with a DOR defined by the following criteria: (i) woman with any of the risk factors for poor ovarian responders and/or (ii) an abnormal ovarian reserve test (i.e., antral follicular count (AFC) <5-7 follicles or AMH <0.5-1.1 ng/ml) (14, 15).

### Clinical Tests

All patients were measured for height and weight. Blood samples were collected from all patients, and serum TSH, free triiodothyronine (FT3), free thyroxine (FT4), and thyroid peroxidation thyroglobulin antibody (thyroid peroxidase antibody, TPOAb), and thyroglobulin antibody (TgAb) were measured using a commercial chemiluminescence immunoassay (Snibe Co.,Ltd., Shenzhen, China; reference range: FT4, 1.00–1.70 ng/dL; TSH, 0.56–4.30 μIU/mL; prolactin, 4.91–29.32 ng/mL). Serum AMH levels were measured using the enzyme-linked immunosorbent assay kit (Guangzhou Kangrun Biotechnology Co., Ltd. Guangzhou, China; and Beckman Coulter Generation II ELISA assay, USA).

### Hormone Measurements

We measured basal serum levels of estradiol (E2), progesterone (P), follicle-stimulating hormone (FSH), and luteinizing hormone (LH) for all patients on the 2nd or 3rd day of their cycle, which we always do before stimulation commencement according to classic protocols. The referral range in our laboratory for basal FSH was 3.5 to 12.5 IU/L, for LH 2.4 to 12.6 IU/L, for E2 0 to 160 pg/mL, and for P4 0 to 1.14 ng/mL. Levels of P4 were also measured on the day of OR. Blood samples were analyzed using chemiluminescent immunoassay (Snibe Co.,Ltd., Shenzhen, China). The size and number of antral follicle (AFC) were measured by Philips HD9 transvaginal ultrasound probe with a 12.0-MHz vginal transducer (Philips Healthcare, Amsterdam, the Netherlands).

### Statistical Analysis

Results were presented as a mean ± standard deviation for variables with normal distribution and as a median and interquartile range for variables for which the distribution was not normal. The normality test of the measurement data adopts the Komogorov-Sminov test. The independent sample t test is used for the comparison between the two groups of data, and the single factor is used for the comparison between the three groups Analysis of variance; count data is expressed as rate (%), using χ2 test or Fisher’s exact probability method. Correlation analysis was performed using the Pearson method. SPSS 19.0 statistical software, P<0.05 is considered statistically significant.

## Results

### Characteristics of Infertile Patients

The FT3 level of the included patients was 0.1~4.76 pg/mL (average 2.72 ± 0.42 pg/mL, of which 5 cases increased, accounting for 1.01%), and the FT4 value was 0.44~ 16.62 ng/dL (average 1.24 ± 1.06 ng/dL, increased 10 cases, accounting for 2.02%), the TSH level was 0.012-8.424 mIU/L (average 2.58 ± 1.37 mIU/L, 18 cases were elevated, accounting for 3.63%). According to the TSH level, they were divided into three groups of <2.5 mIU/L, 2.5~4.0 mIU/L, and ≥4.0 mIU/L. The results showed that there were no significant differences in AMH, basal FSH levels, and AFC among the groups (P>0.05, **Table 1**). Correlation analysis showed that there was no significant correlation between thyroid hormones and ovarian reserve (**Table 2**).

Table 1Comparison of general conditions of patients with different TSH levels.

**Table:** 

TSH	n	Age	BMI (kg/m^2^)	AMH (ng/mL)	E_2_ (pg/mL)	P (ng/mL)
<2.5 mIU/L	288	30.58 ± 4.62	22.46 ± 3.25	5.03 ± 4.17	41.05 ± 15.96	0.70 ± 0.47
2.5- 4 mIU/L	139	30.23 ± 4.38	22.15 ± 3.03	5.13 ± 4.33	38.76 ± 14.97	0.67 ± 0.46
≥4.0 mIU/L	69	29.37 ± 4.13	22.09 ± 2.44	5.57 ± 4.78	40.01 ± 15.38	0.80 ± 0.63
*P*		0.13	0.48	0.64	0.36	0.20

TSH, hyroid-stimulating hormone; FSH, follicle stimulating hormone; LH, luteinizing hormone; E2, estradiol; AMH, Anti-Mullerian hormone; P, progesterone.

**Table:** 

**TSH**	**n**	**FSH (IU/L)**	**LH (IU/L)**	**FSH/LH**	**AFC**	
<2.5 mIU/L	288	8.51 ± 2.69	4.27 ± 2.56	2.51 ± 1.38	16.94 ± 8.10	
2.5- 4 mIU/L	139	8.40 ± 2.36	4.14 ± 2.68	2.89 ± 2.64	17.85 ± 8.26	
≥4.0 mIU/L	69	8.55 ± 2.01	4.44 ± 2.92	2.69 ± 1.66	17.71 ± 7.11	
*P*		0.88	0.74	0.14	0.49	

FSH, follicle stimulating hormone; LH, luteinizing hormone; AFC, antral follicle count.

**Table 2:** Correlation analysis of thyroid hormone and ovarian reserve function.

	Age	AMH (ng/mL)	FSH (IU/L)	LH (IU/L)	FSH/LH	AFC
**TSH (mIU/L)**						
** r**	0.07	0.03	0.01	0.03	0.08	0.03
** *P***	0.13	0.50	0.83	0.54	0.08	0.56
**FT3 (pg/mL)**						
** r**	0.09	0.06	0.04	0.08	0.04	0.00
** *P***	0.06	0.18	0.38	0.08	0.33	0.98
**FT4 (ng/dL)**						
** r**	0.03	0.04	0.02	0.03	0.05	0.06
** *P***	0.57	0.44	0.60	0.51	0.28	0.21

AMH, Anti-Mullerian hormone; FSH, follicle stimulating hormone; LH, luteinizing hormone; AFC, antral follicle count.

### Comparison of Thyroid Related Antibodies and Ovarian Reserve Function

Among 496 patients, the positive rate of TPOAb was 14.52% (72/496), the positive rate of TgAb was 16.94% (84/496), and the positive rate of TPOAb and TgAb was 10.48% (52/496). There were no significant differences in age and AMH among TPOAb (+/- subgroups) (**Table 3**), TgAb (+/- subgroups) (P>0.05, **Table 4**), and TPOAb/TgAb (+/- subgroups) patients (P>0.05, **Table 5**).

**Table 3:** Comparison of TPOAb+ and TPOAb-.

Variables	TPOAb+	TPOAb-	*P*
**Age (years)**	30.28 ± 3.68	30.32 ± 4.62	3.68
**AMH(ng/mL)**	5.22 ± 3.97	5.12 ± 4.36	3.97
**FSH(IU/L)**	8.40 ± 2.34	8.50 ± 2.55	2.34
**FSH/LH**	2.35 ± 1.33	2.69 ± 1.93	1.33
**AFC**	16.29 ± 7.47	17.48 ± 8.10	7.47

FSH, follicle stimulating hormone; LH, luteinizing hormone; AMH, Anti-Mullerian hormone; AFC, antral follicle count.

**Table 4:** Comparison of TgAb+ and TgAb-.

Variables	TgAb+	TgAb-	*P*
**Age (years)**	30.31 ± 4.29	30.32 ± 4.55	4.29
**AMH(ng/mL)**	5.38 ± 4.49	5.08 ± 4.26	4.49
**FSH(IU/L)**	8.62 ± 2.16	8.46 ± 2.58	2.16
**FSH/LH**	2.64 ± 1.50	2.65 ± 1.93	1.5
**AFC**	17.33 ± 7.28	17.30 ± 8.17	7.28

FSH, follicle stimulating hormone; LH, luteinizing hormone; AMH, Anti-Mullerian hormone; AFC, antral follicle count.

**Table 5:** Comparison of TPOAb/TgAb + and TPOAb/TgAb-.

Variables	TPOAb/TgAb+	TPOAb/TgAb-	*P*
**Age (years)**	30.02 ± 3.65	30.35 ± 4.59	3.65
**AMH(ng/mL)**	5.23 ± 3.98	5.12 ± 4.34	3.98
**FSH(IU/L)**	8.52 ± 2.25	8.48 ± 2.55	2.25
**FSH/LH**	2.41 ± 1.45	2.67 ± 1.90	1.45
**AFC**	16.21 ± 5.30	17.43 ± 8.27	5.3

FSH, follicle stimulating hormone; LH, luteinizing hormone; AMH, Anti-Mullerian hormone; AFC, antral follicle count.

### Relationship Between Ovarian Reserve and Thyroid Hormone Levels

The average age of the patients was 30.31 ± 4.50 years old (21-44 years old), and the average AMH was 5.13 ± 4.30 ng/ml (0.08-18ng/ml). According to age, patients were divided into three groups ≤ 29 years old, 30-34 years old, ≥ 35 years old, or grouped according to AMH level, and the differences in thyroid function between the groups were compared. The results showed that no significant differences in age-associated TSH, FT3, and FT4 between the groups (P>0.05, **Table 6**). According to FSH, FSH/LH group comparison, the same result was obtained (P>0.05, **Table 7**). Patients were divided into DOR group and non-DOR group. The results showed that there was still no significant difference in TSH, FT3, and FT4 between the groups (P>0.05, **Table 8**).

**Table 6:** Comparison of thyroid hormone in women of different ages and AMH.

	n	TSH (mIU/L)	FT3 (pg/mL)	FT4 (ng/dL)	TPOAb+	TgAb+
**Age (years)**						
≤ 29	239	2.58 ± 1.35	2.73 ± 0.44	1.20 ± 0.68	13.81 (33/239)	17.15 (41/239)
30-34	175	2.65 ± 1.53	2.72 ± 0.39	1.27 ± 1.35	18.13 (29/160)	16.25 (26/160)
≥ 35	82	2.48 ± 1.11	2.69 ± 0.44	1.29 ± 1.28	10.31 (10/97)	17.53 (17/97)
*F/χ2*		0.48	0.31	2.57	3.16	0.09
*P*		0.62	0.70	0.73	0.21	0.96
**AMH**						
<1.1 ng/mL	61	2.57 ± 1.27	2.66 ± 0.29	1.07 ± 0.18	11.48 (7/61)	16.39 (10/61)
1.1-5 ng/mL	246	2.58 ± 1.44	2.69 ± 0.40	1.22 ± 0.94	15.45 (38/246)	17.48 (43/246)
5-10 ng/mL	119	2.56 ± 1.36	2.81 ± 0.49	1.44 ± 1.69	14.29 (17/119)	12.61 (15/119)
>10 ng/mL	70	2.64 ± 1.27	2.72 ± 0.46	1.12 ± 0.19	14.29 (10/70)	22.86 (16/70)
*F/χ2*		0.051	2.63	2.22	0.63	3.40
*P*		0.985	0.05	0.09	0.89	0.34

TSH, hyroid-stimulating hormone; FT3, free triiodothyronine; FT4, free thyroxine; TPOAb, thyroid peroxidase antibody; TgAb, thyroglobulin antibody.

**Table 7:** Comparison of thyroid hormone in women with different FSH.

	n	TSH (mIU/L)	FT3 (pg/mL)	FT4 (ng/dL)	TPOAb+	TgAb+
**FSH**						
<10 IU/L	386	2.56 ± 1.39	2.72 ± 0.45	1.26 ± 1.20	14.25 (55/386)	17.10 (66/386)
≥10 IU/L	110	2.66 ± 1.33	2.70 ± 0.33	1.16 ± 0.19	15.45 (17/110)	16.36 (18/110)
** *t/χ2***		0.71	0.50	0.86	0.10	0.03
** *P***		0.48	0.62	0.39	0.75	0.86
**FSH/LH**						
<3.6	395	2.55 ± 1.35	2.72 ± 0.44	1.26 ± 1.19	15.19 (60/395)	16.20 (64/395)
≥3.6	101	2.71 ± 1.43	2.72 ± 0.36	1.14 ± 0.21	11.88 (12/101)	19.80 (20/101)
** *t/χ2***		1.05	0.12	1.03	0.40	0.74
** *P***		0.29	0.91	0.130	0.40	0.39

TSH, hyroid-stimulating hormone; FT3, free triiodothyronine; FT4, free thyroxine; TPOAb, thyroid peroxidase antibody; TgAb, thyroglobulin antibody.

**Table 8:** Comparison of thyroid hormone between DOR and non-DOR women.

	n	TSH (mIU/L)	FT3 (pg/mL)	FT4 (ng/dL)	TPOAb+	TgAb+
**DOR**	69	2.44 ± 1.25	2.63 ± 0.31	1.01 ± 0.17	13.04 (9/69)	15.94 (11/69)
**non-DOR**	427	2.60 ± 1.39	2.73 ± 0.43	1.27 ± 1.14	14.75 (63/427)	17.10 (73/427)
***F/χ2***		0.92	1.90	1.50	0.14	0.06
***P***		0.36	0.06	0.13	0.71	0.81

## Discussion

Thyroid hormones are involved in the normal growth, development, and function of many organs, such as the gonads (16–18). TSH, FT3, and FT4 can bind to receptors on ovarian cells and participate in the regulation of ovarian function (19–22). At present, the standard for the cut-off point of TSH level in infertile women is> 2.5 mIU/L or > 4.0-4.5 mIU/L. Previous studies suggest that the risk of adverse pregnancy outcomes is higher in infertile women with over the cut-off point (23–26). However, it is uncertain whether it will affect ovarian reserve. The current evaluation indicators of ovarian reserve include age, AMH, and basic FSH (14, 27). In this study, TSH was grouped into <2.5 mIU/L, 2.5-4 mIU/L, and ≥4.0 mIU/L. The results showed that there were no significant differences in AMH and basal FSH levels among different groups. Further correlation analysis showed no significant difference between TSH, FT3, FT4, AMH, and FSH. This result suggests that there may be no clear correlation between thyroid hormone levels and ovarian reserve in women with infertility (28, 29).

Conflicting results have been reported that the different levels of AMH in women are associated with thyroid dysfunction or confirmed autoimmune thyroid disease (12, 21, 30, 31). The study of thyroid autoimmune antibodies found no significant differences in AMH and basal FSH levels and the number of antral follicles among TPOAb, TgAb, and TPOAb/TgAb groups. It is deduced from this that the ovarian reserve function of infertile women may not be affected by the level of serum thyroid autoimmune antibodies (32). Additionally, subclinical hypothyroidism (SCH), a very common thyroid disorder, is associated with female infertility. SCH is associated with decreased ovarian reserve during later reproductive age. Previous study demonstrated that TPOAb positivity was not associated with ovarian reserve (33). However, it cannot be ruled out that the duration of antibody-positive and the level of antibodies have not affected the hypothalamus-pituitary gland, which false-negative results are obtained from normal physiological activities of the ovarian. Women tend to develop autoimmune diseases more often than men throughout their lifetime. The exact etiology of autoimmune disorders remains unknown; however, it has been postulated that it may be multifactorial and associated with the autoimmune conditions with the X chromosome and X inactivation. The presence of two X chromosomes puts women at a greater risk for developing autoimmune diseases than men (34–36).

The gonadal axis function of women with a reduced ovarian reserve will have a series of changes, which are often clinically manifested as increased basic FSH and increased FSH/LH ratio. It has been reported in the literature that the gonadal axis and the hypothalamus-pituitary-thyroid axis have a mutual regulation effect. Is there a corresponding change in thyroid function when the ovarian reserve declines? At present, the diagnostic criteria of ovarian reserve are not uniform. The often concerned indicators are age ≥35 years, AMH<0.5~1.1 ng/ml, basic FSH>10 IU/L, and FSH/LH≥3.6 (14, 27). In this study, the patients were divided into three groups according to age ≤ 29 years old, 30-34 years old, and ≥ 35 years old, or according to AMH levels as <1.1 ng/ml, 1.1-5 ng/ml, 5-10 ng/ml and 10 ng/ml four groups, or according to the basic FSH level, they were divided into <10 IU/L, ≥10 IU/L two groups. We compared the differences in thyroid function between the groups and found that no matter what method is used for grouping, there was no significant correlation in TSH, FT3, FT4, TPOAb positive rate, and TgAb positive rate. This result indicates that any single indicator for evaluating ovarian reserve may not significantly correlate with thyroid function. In order to further clarify the relationship between the ovarian reserve and thyroid function, we analyzed the effect of DOR in the correlation. The results showed that there was still no significant difference in thyroid function between the DOR group and the non-DOR group. The epidemiological study reported by Colella M et al. found that the prevalence of both dominant and subclinical hypothyroidism among women with genetic factors of hypothyroidism was significantly higher (22). There was no significant difference in thyroid function between DOR women in this study and non-DOR women. It may be related that the included patients did not have a classification related to genetic factors or related to the small sample size.

## Conclusions

This study found no significant correlation between the ovarian reserve and thyroid function-related indicators in women with infertility. However, this study has the following some limitations. 1) The sample size is small, especially the number of thyroid antibody-positive cases.The study will be further expanded in the later period to analyze the potential relevance in depth. 2) The relationship between the duration of abnormal thyroid function and ovarian reserve is required to further analysis. Future study is required to investigate the underlying molecular mechanisms regulating the diminished ovarian reserve in women with thyroid disorder.

## Data Availability Statement

The raw data supporting the conclusions of this article will be made available by the authors, without undue reservation.

## Ethics Statement

The studies involving human participants were reviewed and approved by Ethics Committee of Affiliated Hospital of Southwest Medical University. The patients/participants provided their written informed consent to participate in this study.

## Author Contributions

JW and Y-FL conceived and designed the study. JW, Y-JZ, MW, M-QT, and Y-FL collected patient data. Y-FL reviewed and edited the manuscript. All authors contributed to the article and approved the submitted version.

## Funding

This study was supported in part by grant from the Doctoral Research Initiation Fund of the Affiliated Hospital of Southwest Medical University (NO.19084) and Sichuan Provincial Administration of Traditional Chinese Medicine (No. 2021MS488).

## Conflict of Interest

The authors declare that the research was conducted in the absence of any commercial or financial relationships that could be construed as a potential conflict of interest.

## Publisher’s Note

All claims expressed in this article are solely those of the authors and do not necessarily represent those of their affiliated organizations, or those of the publisher, the editors and the reviewers. Any product that may be evaluated in this article, or claim that may be made by its manufacturer, is not guaranteed or endorsed by the publisher.
